# 4-Chlorophenol adsorption from water solutions by activated carbon functionalized with amine groups: response surface method and artificial neural networks

**DOI:** 10.1038/s41598-023-35117-4

**Published:** 2023-05-15

**Authors:** Moslem Tazik, Mohammad Hadi Dehghani, Kamyar Yaghmaeian, Shahrokh Nazmara, Mehdi Salari, Amir Hossein Mahvi, Simin Nasseri, Hamed Soleimani, Rama Rao Karri

**Affiliations:** 1 Department of Environmental Health Engineering, School of Public Health, Tehran University of Medical Sciences, Tehran, Iran; 2 Institute for Environmental Research, Center for Solid Waste Research, Tehran University of Medical Sciences, Tehran, Iran; 3 Department of Environmental Health Engineering, School of Public Health, Sabzevar University of Medical Sciences, Sabzevar, Iran; 4 Student’s Scientific Research Center, Tehran University of Medical Sciences, Tehran, Iran; 5 Petroleum and Chemical Engineering, Faculty of Engineering, Universiti Teknologi Brunei, Bandar Seri Begawan, BE1410 Brunei Darussalam

**Keywords:** Environmental sciences, Planetary science

## Abstract

4-Chlorophenol pollution is a significant environmental concern. In this study, powdered activated carbon modified with amine groups is synthesized and investigated its efficiency in removing 4-chlorophenols from aqueous environments. Response surface methodology (RSM) and central composite design (CCD) were used to investigate the effect of different parameters, including pH, contact time, adsorbent dosage, and initial 4-chlorophenol concentration, on 4-chlorophenol removal efficiency. The RSM-CCD approach was implemented in R software to design and analyze the experiments. The statistical analysis of variance (ANOVA) was used to describe the roles of effecting parameters on response. Isotherm and kinetic studies were done with three Langmuir, Freundlich, and Temkin isotherm models and four pseudo-first-order, pseudo-second-order, Elovich, and intraparticle kinetic models in both linear and non-linear forms. The synthesized adsorbent was characterized using X-ray diffraction (XRD), Fourier transforms infrared spectroscopy (FTIR), and scanning electron microscopy (SEM) analyses. The results showed that the synthesized modified activated carbon had a maximum adsorption capacity of 316.1 mg/g and exhibited high efficiency in removing 4-chlorophenols. The optimal conditions for the highest removal efficiency were an adsorbent dosage of 0.55 g/L, contact time of 35 min, initial concentration of 4-chlorophenol of 110 mg/L, and pH of 3. The thermodynamic study indicated that the adsorption process was exothermic and spontaneous. The synthesized adsorbent also showed excellent reusability even after five successive cycles. These findings demonstrate the potential of modified activated carbon as an effective method for removing 4-chlorophenols from aqueous environments and contributing to developing sustainable and efficient water treatment technologies.

## Introduction

Phenol and its derivatives, including chlorophenols containing synthetic organic compounds, are ubiquitously found in the effluents of industries such as petrochemical, coal production, rubber, plastic, steel, and aluminium. These compounds are important in terms of environmental and health effects due to their relative stability in the environment, resistance to biological decomposition, ability to dissolve in water, and their carcinogenic nature^1^. Chlorophenol compounds can accumulate in the sediments and food chain. These compounds are entered into water resources and remain in the environment long. 4-Chlorophenol (C_6_H_5_ClO) is one type of chlorophenol widely used in petrochemical, insecticide, herbicide, industrial dyes, and pharmaceutical industries^2^.

4-Chlorophenol causes skin and eye irritation through skin contact and short-term inhalation; long-term exposure to 4-chlorophenol imposes severe damage to the liver, kidney, and central nervous system^3^. The International Agency for Research on Cancer (ICRA) classified chlorophenols in group B2^4^, and the US Environmental Protection Agency has set a phenol threshold of less than 1 part per billion (ppb) in surface water as a treatment level^5^. 4-Chlorophenol, due to its stability against mineralization, is difficult to treat compared to other chlorophenols^6^.

Over recent years, a variety of technologies have been used to remove the 4-chlorophenols from drinking water and wastewater, including chemical oxidation^7^, biological purification^5^, and wet oxidation^8^. Oxidation methods often produce dangerous side products and significant amounts of solid waste; they need high disposal and regeneration costs^9^. The efficiency of biological treatment processes for phenolic wastewater is usually unsatisfactory due to chlorophenols’ toxic effects on microorganisms, biological systems' inability to withstand shock loads, and long retention times^9^. Chemical methods also require high investment and increase the load of soluble chemicals in the wastewater^10,11^. Thermal methods release harmful side products from incomplete combustion, such as dioxins and fumes, into the air^12^. One of the most common methods developed recently to remove these compounds is activated carbon which is efficient in the surface adsorption method of pollutants^13^.

Surface adsorption with activated carbon, due to its simplicity and ease of operation^14^, cheapness, lack of sludge production^9^, and the ability to recycle adsorbed materials^15^, is considered an acceptable method in water and wastewater treatment. However, further studies still need to improve the efficiency of these types of absorbents through various modification methods^16^. The use of functional groups has been widely considered due to their low cost, simplicity, appropriate separation speed, and high efficiency^17^. In this regard, Mahaniyya et al.^18^ employed activated carbon modified with amine groups to adsorb copper from aqueous solutions. The authors reported that the presence of amine groups increases the efficiency of copper adsorption on modified activated carbon (removal efficiency). In addition, according to the literature, the modification of activated carbon with amine groups to adsorb 4,2 dichlorophenol^19^ and the use of aminated biosorbents to remove pentachlorophenol^20^ confirm the effectiveness of modification methods in increasing the removal efficiency of the pollutants.

Therefore, as the functionalization of the nanoparticle’s surface is widely used in catalytic applications, this type of adsorbent is welcomed in removing many organic compounds. Synthesis of these adsorbents can be beneficial due to some advantages such as increased specific area and, consequently, the increased absorption capacity of the adsorbent, shortening the reaction time, and increasing efficiency and stability^21^.

According to the limited research on using modified activated carbons to remove 4-chlorophenols, the present study was developed to synthesize the powdered activated carbon adsorbents functionalized with amine groups and investigate their efficiency in the removal of 4-chlorophenols from aqueous environments. The present study’s additional aim was to investigate the role of influencing parameters (pH, adsorbent dose, contact time, and initial 4-chlorophenols concentration) on 4-chlorophenols removal efficiency. The RSM package and the Central Composite Design (CCD) approach determined the optimal condition. Modelling and optimal process condition were performed using Response Surface Method in R software and Artificial Neural Networks. The combination of RSM and ANNs can provide a powerful tool for optimizing the adsorption conditions and predicting the adsorption performance of the modified activated carbon. This approach can lead to more efficient and cost-effective removal of 4-Chlorophenol from water environments, significantly contributing to water treatment and environmental remediation. The resulting models were then further applied to optimize the 4-chlorophenols adsorption, with different parameters leading to the maximization of efficiency.

## Materials and methods

### Chemicals

All chemicals used in this study were of analytical type. The chemicals used in this study, including 4-chlorophenols, powdered activated carbon, caustic soda, dimethylformamide, and hydrochloric acid, were obtained from Merck (Germany). HCl (1 N) and NaOH (0.1 N) were used to adjust the pH, and deionized water (Milli Q Millipore 18.2 MΩ cm^−1^ conductivity) was used for the preparation of all solutions.

#### The 4-chlorophenol (stock solution and chemical properties)

The stock solution was prepared by dissolving 1000 mg of 4-chlorophenol solid powder in 1 L of distilled water. The concentration of 4-chlorophenol was measured by a UV visible spectrophotometer, model DR 5000 ((Hatch-Lange, England), at a wavelength of 280 nm^20^. 4-Chlorophenol is a chlorinated aromatic compound with the chemical formula C_6_H_5_ClO. It is a colourless to white crystalline solid soluble in water and organic solvents. 4-Chlorophenol is a toxic and persistent environmental pollutant commonly found in industrial wastewater and agricultural runoff. The United States Environmental Protection Agency (EPA) classifies it as a priority pollutant due to its potential health and environmental effects^22^.

### Adsorbent properties and preparation

Activated carbon is a highly porous form of carbon with a large surface area and high microporosity, making it an effective adsorbent for a wide range of organic and inorganic compounds. It can be functionalized with amine groups by treating it with amines that react with the activated carbon’s surface functional groups to form amine-functionalized carbon. The amine groups on the surface of the activated carbon can interact with the 4-chlorophenol molecules through hydrogen bonding and electrostatic interactions, leading to the adsorption of 4-chlorophenol from aqueous environments. The amine-functionalized activated carbon has been shown to have higher adsorption capacity and selectivity for 4-chlorophenol than non-functionalized activated carbon. The procedure of linking modified amino groups was used to convert PAC into an ion exchanger. The synthesis steps of the ion exchanger were carried out in two separate parts as follows.

#### Preparation of intermediate amine solution

To prepare the intermediate amine solution, 39 mL epichlorohydrin and 76 mL trimethylamine was added to 75 mL dimethylformamide (DMF) (as a solvent) at 80 °C for 120 min in an evaporator^23^.

#### MAC synthesis

To synthesize MAC, 10 gr PAC and 30 mL intermediate amine solution prepared in the previous step and 10 mL pyridine (as a process catalyst) were placed in the evaporator for 2 h at 60 °C. After filtration, the resulting product was washed with 500 mL of 0.1 M HCl solution. 0.1 M NaOH solution was used to remove the remaining chemicals. At the end of MAC, a sufficient amount was washed with double distilled water until the resulting effluent reached neutral pH, and the final product was dried at 50 °C for 24 h. The obtained sample was modified activated carbon (MAC) and used as an adsorbent^23,24^.

### Adsorbent characterization

The modified MAC properties were characterized by XRD analysis (X-Ray Diffractometer model PW1800, The Netherlands) at the wavelength of 1.541 angstroms (0.1541 nm) in the range of Cu radiation, voltage 40 kV, and current 30 mA, and scan range (2Ɵ) from 10 to 80 degrees, was tested. The surface structure and morphology of natural and modified adsorbents were examined by scanning electron microscopy (SEM) using a Philips XL30 microscope with an accelerating voltage of 15 kV (Philips XL30, Philips Netherlands). Fourier identified PAC and MAC surface functional groups transform infrared spectroscopy (FTIR, spectrum 3, PerkinElmer, America) in the 400–4000 cm^−1^.

### Experimental design and optimization

#### Response surface methodology (RSM)

The effect of influencing variables pH, retention time (min), adsorbent dose (g/L), and initial concentration (mg/L) as input variables on the efficiency of powdered activated carbon functionalized with amine groups in removing of 4-Chlorophenol were investigated as a response variable. The RSM package and CCD approach method determined the optimal condition using R software version 3.3.2.0 (2016-10-31)^25^. The predictor variables were standardized, and then the hierarchical RSM model with all first-order terms, all pure quadratic terms, and all two-way interactions were fitted. The levels of influencing parameters were as follows: initial 4-Chlorophenol concentration (20–200 mg/L), contact time (10–60 min), pH (3–11), and adsorbent dosage (0.1–1 g/L). The variables used in the present study and their levels are given in Table 1.

**Table 1:** The actual and coded values of independent variables.

Variables	Unit	Range and level	
		− α (− 1)	+ α (+ 1)
pH	–	3	11
Contact time (X_3_),	min	10	60
Adsorbent dosage	g/L	0.1	1
4-Chlorophenol concentration	Mg/L	20	200

RSM includes a set of experimental techniques used to investigate the relationship between experimental factors and measured responses, and the determined responses correspond to one or more selected items. This technique first predicts the relationship between the dependent variables (response) and the independent variables. In the next step, the behaviour of the variables and the response are specified using Eq. (1)^26^:

1 $$\mathrm{y}={\upbeta }_{0}+\sum \limits_{\mathrm{i}=1}^{\mathrm{k}}{\upbeta }_{\mathrm{i}}{\mathrm{x}}_{\mathrm{i}}+\sum \limits_{\mathrm{i}=1}^{\mathrm{k}}{\upbeta }_{\mathrm{ii}}{\mathrm{x}}_{\mathrm{i}}^{2}+\sum \limits_{1\le \mathrm{i}\le \mathrm{j}}^{\mathrm{k}}{\upbeta }_{\mathrm{ij}}{\mathrm{x}}_{\mathrm{i}}{\mathrm{x}}_{\mathrm{j}}+\upvarepsilon ,$$

where k is the number of input variables, β_0_ is the constant term, β_i_ the coefficients of the linear variables, x_i_ is the input variables, β_ii_ the coefficients of the quadratic parameter, β_ij_ the interaction coefficients between input variables, and $\upvarepsilon$ the error associated with the experiments. The experimental design was performed by R software using the CCD method, which included 44 experiments (Table 2).

**Table 2:** Experimental conditions and results of central composite design.

Run	X_1_	X_2_	X_3_	X_4_	Pre. (RSM) (%)	A.C. (%)	Run	X_1_	X_2_	X_3_	X_4_	Pre. (RSM) (%)	Pre. (ANN) (%)	AC. (%)
1	7	35	0.55	110	83.520	87.277	23	5.45	25.32	0.72	144.86	79.141	75.26	76.337
2	7	35	0.55	110	83.520	89.855	24	8.55	44.68	0.72	144.86	71.118	70.17	70.169
3	8.55	44.68	0.37	144.86	38.125	40.313	25	7	10	0.55	110	42.665	41.82	41.824
4	7	35	0.55	110	83.520	92.353	26	7	35	0.55	110	83.520	84.65	77.967
5	5.45	25.32	0.72	75.14	96.867	92.569	27	7	35	1	110	87.736	90.84	90.836
6	5.45	44.68	0.72	75.14	96.546	91.264	28	7	35	0.55	110	83.520	84.65	80.837
7	8.55	25.32	0.37	75.14	43.826	46.876	29	7	35	0.55	20	60.434	64.51	63.126
8	8.55	44.68	0.72	75.14	65.569	65.436	30	7	35	0.55	110	83.520	84.65	80.527
9	8.55	25.32	0.72	144.86	49.748	50.273	31	7	35	0.55	200	28.110	29.28	29.284
10	7	35	0.55	110	83.520	76.255	32	7	35	0.55	110	83.520	84.65	83.836
11	7	35	0.55	110	83.520	86.247	33	11	35	0.55	110	14.224	25.76	10.029
12	7	35	0.55	110	83.520	85.374	34	7	35	0.55	110	83.520	84.65	84.625
13	5.45	44.68	0.37	75.14	72.941	70.305	35	7	35	0.55	110	83.520	84.65	80.164
14	8.55	25.32	0.37	144.86	14.467	16.600	36	7	35	0.55	110	83.520	84.65	81.839
15	5.45	44.68	0.37	144.86	63.641	61.886	37	7	35	0.55	110	83.520	84.65	83.873
16	5.45	25.32	0.37	144.86	40.006	38.027	38	7	60	0.55	110	72.717	75.97	75.973
17	7	35	0.55	110	83.520	82.893	39	7	35	0.55	110	83.520	84.65	79.935
18	5.45	25.32	0.37	75.14	70.973	68.774	40	7	35	0.55	110	83.520	84.65	84.456
19	7	35	0.55	110	83.520	81.298	41	3	35	0.55	110	87.290	95.35	**95.352**
20	5.45	44.68	0.72	144.86	94..487	95.326	42	7	35	0.55	110	83.520	84.65	84.368
21	8.55	25.32	0.72	75.14	65.867	64.473	43	7	35	0.1	110	13.487	13.58	12.824
22	8.55	44.68	0.37	75.14	45.817	45.473	44	7	35	0.55	110	83.520	84.65	92.837

#### Artificial neural network (ANN)

ANN method was the other approach used for modelling and predicting 4-Chlorophenol based on the dataset presented in Table 2. ANN approach takes inspiration from important human nervous system functions to approximate complex system behaviours. Also, it has a remarkable capability in classification, training, and data validation. In the current study, the flexible mathematical structure of the Multi-layer perceptron method with a Levenberg–Marquardt backpropagation training algorithm (Fig. 4) was established for the ANN model to unveil complicated non-linear relationships.

As seen in Fig. 4, ANN architecture was made of three layers: one input layer with five neurons, one hidden layer, and one output layer with one neuron. The number of neurons in the hidden layer was optimized upon the lowest MSE and largest R^2^ outcomes of the fitting between the experimental and prediction dataset. These statistical measures were explored to try and error test in different numbers of 1 to 20 neurons. Transfer functions in hidden and output layers were selected as Sigmoid and purline functions. The dataset tabulated in Table 2 is grouped randomly into three sets, including training (70%), validating (15%), and testing (15%) data. Also, all the data were normalized, ranging from 0.1 to 0.9, to improve the training data quality and minimize computational problems. Equation (2) is used for the normalization

2 $${y}_{i}=0.1+0.8 \times \frac{{x}_{i}- {x}_{min}}{{x}_{max}- {x}_{min}},$$

where y_i_ is the normalized output value, and x_max_ and x_min_, denote the maximum and minimum values of input variables, respectively. ANN model was prepared using Neural Network Toolbox (MATLAB, 2013).

In addition to the CCD-RSM approach, independent variables were optimized using the ANN-GA technique. The GA optimization approach was made in the MATLAB GA toolbox. To this end, the equation established from the ANN model is written in a script file. In the MATLAB GA toolbox, the high and low levels of the process parameters, as observed in Table 2, were added to the lower and upper bound, and default settings were retained unchanged unless specified. Finally, the optimization results obtained from both approaches (CCD-RSM and ANN-GA) were compared with the control experiments to find the best approach.

### Measuring the 4-chlorophenol concentration

The calibration of the spectrophotometer was a crucial step in accurately measuring the concentration of 4-chlorophenol in the study. Using a stock solution with a concentration range of 20–200 mg/L and repeating all concentrations three times ensured the accuracy of the calibration. The resulting standard curve had a high coefficient of determination (R^2^ = 0.999), indicating a strong correlation between the adsorption and concentration of 4-chlorophenol. The accurate measurement of 4-chlorophenol concentration was essential in determining the efficiency of the modified activated carbon in removing 4-chlorophenol from aqueous environments^22^.

### Adsorption experiment

#### Adsorption isotherm experiments

To check the adsorption isotherm, 0.77 g of the MAC was added to different concentrations (mg/L) of 4-chlorophenol (20, 10, 110, 75, 144, 200, 220) at the optimal pH and for 43 min in a shaker at 150 rpm. Finally, the final concentration of the pollutant was measured by a spectrophotometer. Equation (3)^24,27^ was used to determine the equilibrium adsorption capacity (qe) of 4-chlorophenol MAC, expressed in terms of mg (4-CP)/g MAC.

3 $${\mathrm{q}}_{e}=\frac{\left({C}_{0}-{C}_{e}\right)}{{x}_{ads}}\times v,$$

where q_e_ is the amount of adsorbed 4-chlorophenol (mg/g), C_0_ is the initial concentration of 4-chlorophenol (mg/L), C_e_ is the final concentration of 4-chlorophenol at time t, V is the volume of the solution (L), and x_ads_ is a mass of adsorbent (g). Table 3 shows the isotherm relationships used in the study. This section used linear relationships to fit isotherm models with experimental findings^28^.

**Table 3:** The linear and non-linear equations of adsorption isotherm models.

Isotherm	Linear equation	Non-linear equation	Parameters
Langmuir	$\frac{{C_{e} }}{{q_{e} }} = \left( {\frac{1}{{K_{L} q_{m} }}} \right) + \left( {\frac{1}{{q_{m} }}} \right)C_{e}$	$q_{e} = \frac{{q_{m} K_{a} C_{e} }}{{1 + K_{a} C_{e} }}$	$$\begin{gathered} q_{m} = \frac{1}{slope} \hfill \\ K_{L} = \frac{1}{{intercept \times q_{m} }} \hfill \\ \end{gathered}$$
Freundlich	$\log (qe) = \log \left( {K_{f} } \right) + \left( \frac{1}{n} \right)\log (C_{e} )$	$q_{e} = K_{f} \times C_{e}^{\frac{1}{n}}$	$$\begin{gathered} K_{f} = \exp (intercept) \hfill \\ Slope = \frac{1}{n} \hfill \\ \end{gathered}$$
Temkin	$q_{e} = B_{T} \ln A_{T} + B_{T} \ln C_{e}$	$q_{e} = \frac{RT}{b}\ln \left( {A_{T} C_{e} } \right)$	B_T=_ slope ln A_T_ = intercept/B.T

The adsorption tendency of the pollutant to the adsorbent can be evaluated using a dimensionless parameter (R_L_) called the balance factor, derived from the Langmuir equation (Eq. 4)^21^:

4 $${\mathrm{R}}_{\mathrm{L }= \frac{1}{1+({\mathrm{K}}_{\mathrm{L}}+{\mathrm{C}}_{\mathrm{e})}}}$$

Ci is the pollutant’s initial concentration (mg/L), and K_L_ is the Langmuir constant (L/mg). R_L_ values can be interpreted based on the data in Table 4^18^.

**Table 4:** Langmuir isotherm constant parameter, R_L._

R_L_ value	Type of isotherm
R_.L._ > 1	Unfavourable
R_L_ = 1	Linear
R_L_ = 0	Irreversible
0 < R_L_. < 1	Favourable

### Adsorption kinetic experiments

Four pseudo-first-order, pseudo-second-order, and pseudo-second-order kinetic models were used to determine MAC's kinetic coefficients of 4-chlorophenol Removal (Table 5)^26,29^.

**Table 5:** The linear and non-linear forms of adsorption kinetic models.

Kinetic	Linear equation	Non-linear equation	Parameters
Pseudo-first-order	$\log \left( {q_{e} - q_{t} } \right) = \log \left( {q_{e} } \right) - \frac{{k_{1} }}{2.303}t$	$\frac{{dq_{t} }}{dt} = k_{1} \left( {q_{e} - q_{t} } \right)$	*k*_1_ = slope *q*_e_ = intercept
Pseudo-second-order	$\frac{t}{{q_{t} }} = \left( {\frac{1}{{k_{2} q_{e}^{2} }}} \right) + \left( {\frac{1}{{q_{e} }}} \right)t$	$\frac{{dq_{t} }}{dt} = k_{2} \left( {q_{e} - q_{t} } \right)^{2}$	1/q_e_ = Slope 1/(k_2_q_e_^2^) = Intercept
Elovich	$q_{e} = \left( {\frac{1}{\beta }} \right)\ln \left( {\alpha \beta } \right) + \left( {\frac{1}{\beta }} \right)\ln t$	$\frac{{dq_{t} }}{dt} = \alpha \exp \left( { - \beta q_{t} } \right)$	1/β = Slope 1/β × ln (αβ) = Intercept
Intraparticle diffusion	$q_{t} = K_{dif} t^{0.5} + C$	*–*	C = Intercept K_dif_ = Slope

### Thermodynamic study

Gibbs free energy (∆G^0^), enthalpy variations (∆H^0^), and entropy (∆S^0^) resulting from the adsorption were calculated by thermodynamic study. These parameters provide important information about inherent energetic changes, nature, and the spontaneity of the adsorption reaction^11,30^. The thermodynamic distribution coefficient (K_d_) is required to determine the thermodynamic parameters obtained by the following^31^:

5 $$Q={Q}_{max}\frac{{\mathrm{K}}_{\mathrm{d}}{[\mathrm{b}]}^{n}}{1+ {\mathrm{K}}_{\mathrm{d}}{[\mathrm{b}]}^{n}}.$$

Here *Q*_max_ (mg/g) and *b* (mg/L) stand for the adsorption capacity of MAC and the concentration of 4-chlorophenol at the equilibrium, respectively. And *n* assigns to a constant. Equations (6) and (7) provide the ∆G°, ∆H°, and ∆S° parameters^32^.

6 $$\Delta {G}^{^\circ }=-RT {\text{ln}} {K}_{d},$$

7 $$\mathrm{ln}{K}_{d}=\frac{\Delta {S}^{^\circ }}{R}-\frac{\Delta {H}^{^\circ }}{RT},$$

where *R* is devoted to the gas constant (8.314 J mol^−1^ K^−1^), and *T* denotes the reaction solution temperature (°K), which differed in the range of 288–328 °K.

### Statistical analysis

This study used R software, version 3.3.2.0 (2016-10-31), to determine the number of experimental tests, RSM method, modelling, and optimization^20^.

## Results and discussion

### Adsorbent characteristics

#### XRD analysis

**Figure 1:**
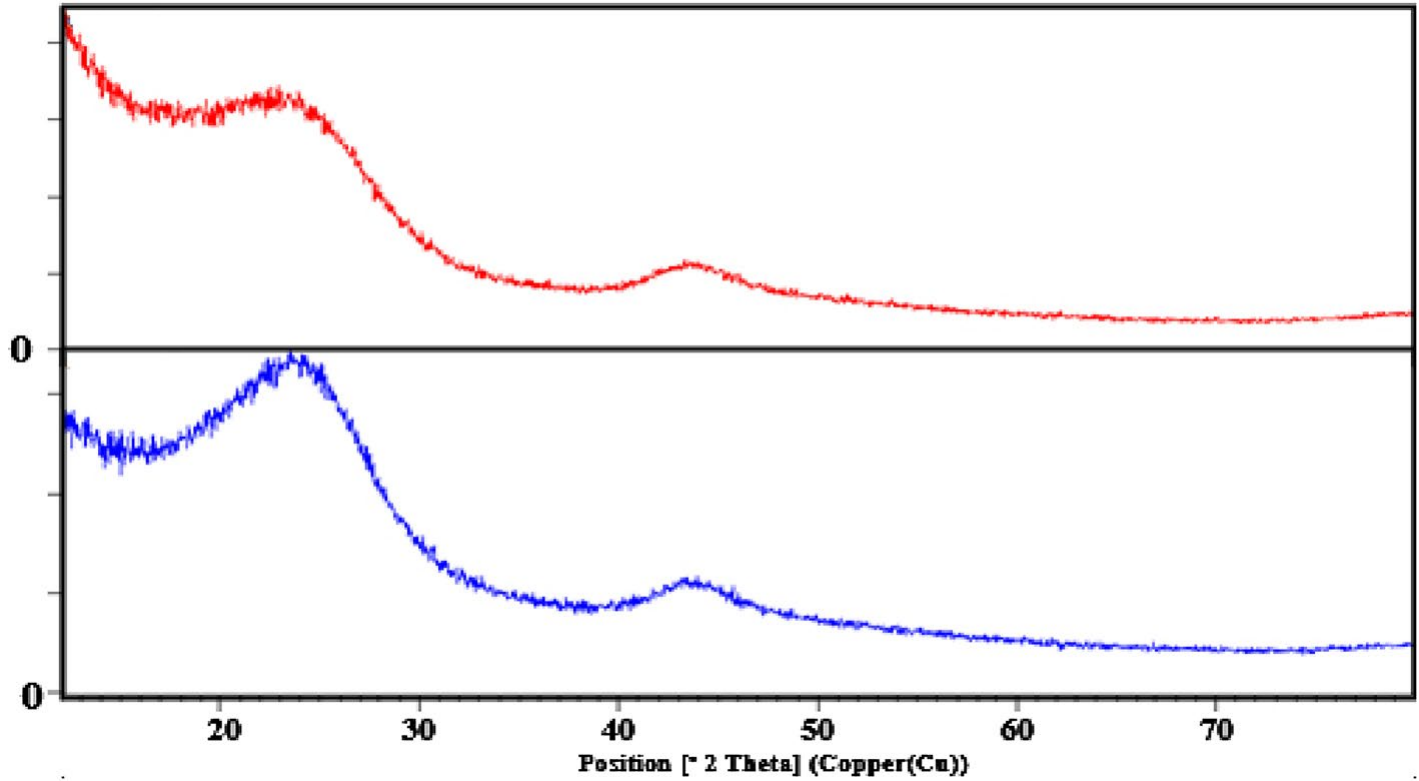
XRD analysis for PAC (Red) and MAC (Blue).

X-ray diffraction (XRD) analysis was used to investigate the chemical compounds on both powdered activated carbon (PAC) and modified activated carbon with amine groups (MAC). XRD analysis was performed at *Ɵ* = 10–109° at 25 °C with Cu radiation, a voltage of 40 kW, and a current of 30 mA, scanning range of 5 to 65°. Figure 1 shows the XRD pattern of PAC and MAC powder.

According to Fig. 1, peaks at 2*Ɵ* equal to 24.59 and 43.64 indicated slight changes in the peaks and bandwidth of the crystal structure of MAC compared to the adsorbent before modification. It shows that the surface of the adsorbent has been modified, and carbon nanoparticles active have a pure and crystalline structure.

#### SEM analysis

**Figure 2:**
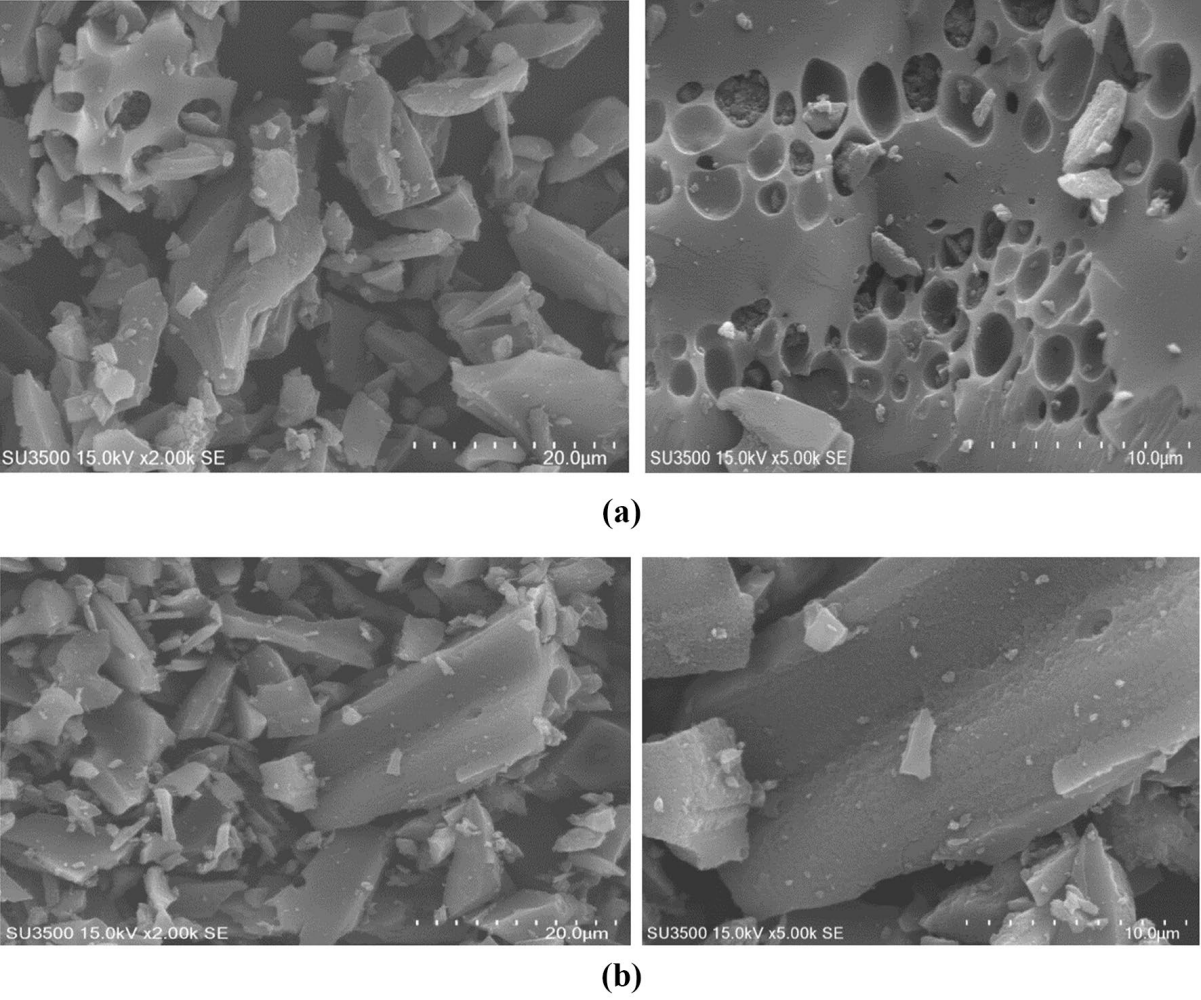
SEM (**a**) Raw activated carbon. (**b**) modified activated carbon (MAC).

As can be seen, the MAC Surface (Fig. 2b) is softer and more velvety than PAC (2a), indicating the noticeable and significant effect of surface modification with amine groups. The natural adsorbent (Fig. 2a) has uniform surfaces and is much stronger than the modified absorbent, smooth, and without corrosion. While in the case of MAC modified with amine groups, the surface is softer and has fragment pieces and porosity. It is observed that it is caused by the effect of amine groups on the absorbent structure^33,34^. Surface modification with amine groups on the MAC Surface led to higher removal efficiency than the PAC. This could be because the amine groups on the MAC Surface provided additional active adsorption sites or enhanced the chemical interactions between the adsorbent and the adsorbate. So, while the surface of the MAC Surface may have been smoother, the presence of the amine groups made it a more effective adsorbent overall^26,35^.

#### FTIR analysis

**Figure 3:**
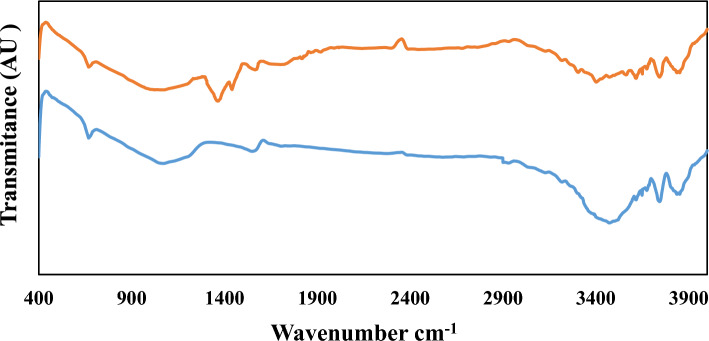
FTIR analyses for PAC (Blue) and MAC) Orange.

Infrared spectroscopy is performed based on radiation adsorption and investigation of vibrational transitions of molecules and polyatomic ions. Figure 3 shows the FTIR spectrum of PAC and MAC powder.

FTIR technique is commonly used as a powerful and developed method to determine the structure and measurement of chemical species. In addition, this method is mainly used to identify organic compounds because the spectra of these compounds are usually complex and have a large number of maximum and minimum peaks, which can be used for comparative purposes^36^. When the amine groups introduce to the activated carbon surface, creating the MAC, you should expect new peaks in the FTIR spectra. These peaks correspond to the amine functional groups (NH2) grafted onto the carbon surface. You may see peaks around 3300–3500 cm^-1^ (N–H stretching vibrations) and 1300–1600 cm^−1^ (C–N bending vibrations). As seen in Fig. 3, the peak created around the 1364 cm^−1^ band is related to the presence of amine groups (C–N stretching vibrations) coated on the active carbon. Therefore, it can be said that amine groups are well covered in activated carbon^33,34^.

### RSM and ANN analysis

The RSM and CCD approach was performed to find the optimal points^26^. This study used the RSM-CCD approach and ANOVA test to validate the model. Table 6 summarizes the results obtained from the statistical analysis of operational parameters. The predicted values obtained from the model are also given in this table. The model’s results showed that the presented model is not much different from the actual results obtained; the R^2^ values obtained from the correlation between the obtained results and the predicted values for 4-CP adsorption were 0.9738. In addition, an ANOVA test was used to evaluate model regression and interaction effects for removing 4-chlorophenol from water environments. The coefficients of the coded variables indicated that except for the parameters ((adsorbent concentration- time), (adsorbent concentration -pH), (time- pH)), all the components of the model have a P-Value less than 0.05, which means that most of the model components are statistically significant^37^.

**Table 6:** The regression analysis for 4-CP removal.

Factor	Estimate	Std. error	t value	Pr( >|t|)	P-value
Intercept	83.2731223	0.977795	85.1642	< 2.2e−16	< 0.001
pH	− 11.694040	0.6910103	− 16.9231	< 2.2e−16	< 0.001
Time	4.9088300	0.6910103	7.1038	8.130e−08	< 0.001
Ads	11.7721032	0.6910365	17.0354	< 2.2e−16	< 0.001
Conc 4-CP	− 5.1559450	0.6910103	− 7.4615	3.188e−08	< 0.001
Time: Conc	3.6955372	0.7728110	4.7819	4.649e−05	< 0.001
Time: Ads	− 0.3787663	0.7713741	− 0.4910	0.627102	
Ads: Conc	2.2485559	0.7712534	2.9155	0.006783	< 0.01
pH: Ads	− 0.6940979	0.7711027	− 0.9001	0.375462	
pH: time	0.0040712	0.7726599	0.0053	0.995832	
(pH)^2^	− 3.3059656	0.3357259	− 9.8472	9.348e−11	< 0.001
(Time)^2^	− 2.6678854	0.3354778	− 7.9525	9.037e−09	< 0.001
(Ads)^2^	− 3.4035277	0.3369254	− 10.1017	5.243e−11	< 0.001
(Conc)^2^	− 3.9679952	0.3355880	− 11.8240	1.296e−12	< 0.001

Table 7 shows the analysis of variance (ANOVA) of the first-order and second-order model and the interaction effects of removing 4-chlorophenol from the water environment.

**Table 7:** ANOVA test for CCD modelling and results of process optimization.

Probability (> F)	DF	Sum sq	Mean sq	F value	Probability (> F)
First-order response	4	14,151.5	3537.9	172.3158	< 2.2e−16
Two-way interaction	6	668.2	111.4	5.4240	0.0007321
Pure quadratic response	4	7296.6	1824.2	88.8478	7.658e−16
Residuals	29	595.4	20.5	–	–
Lack of fit	10	232.4	23.2	1.2168	**0.3409785**
Pure error	19	363.0	19.1	–	–

Lack of fit = 0.3409875, Multiple R^2^ = 0.9738, Adjusted R^2^ = 0.9611, F-statistic = 76.94 on 14 and 29 DF, P-value = 2.2e−16.

Significant values are in bold.

The R^2^ value of the predicted model was found to be 0.9738. This value shows that the predicted model is meaningful and can be used to show the relationship between the independent variables and the response^24^. In addition, the adjusted R^2^ value for this model was 0.9611. In other words, 0.9611% of the variable changes of 4-chlorophenol removal can be justified by using this model.

The ANOVA analysis indicated that P-values related to first-order terms (FO), pure quadratic (PQ) terms, and two-way interactions (TWI) are all < 0.05. In addition, the P-value of the lack of fit test is 0.3409875, and it is > 0.05, which means that the model with all first-order terms, all pure quadratic terms, and all two-way interactions fit the data overall well. In addition, the adjusted R^2^ value for this model was equal to 96.11% (Table 7), indicating that 96.11% of the variable changes of 4-chlorophenol removal can be justified by using this model. The difference between R^2^ and adjusted R^2^ was 0.1, which indicates good model quality^19^. The quadratic equation related to the CCD model for the absorption of 4-chlorophenol on MAC can be defined as Eq. (8):

8 $${\text{R}} = 83.27312 - 11.68966({\text{pH}}) + 4.91121({\text{Time}}) + 11.77210({\text{Ads}}) - 5.15595({\text{Conc}}) - 3.30597({\text{pH}})^{2} - 2.66789({\text{Time}})^{2} - 3.40353({\text{Ads}})^{2} - 3.96800({\text{Conc}})^{2} + 3.69554({\text{Conc}}) \times ({\text{Time}}) + 2.24856({\text{Conc}}) \times ({\text{Ads}}).$$

### ANN model

**Figure 4:**
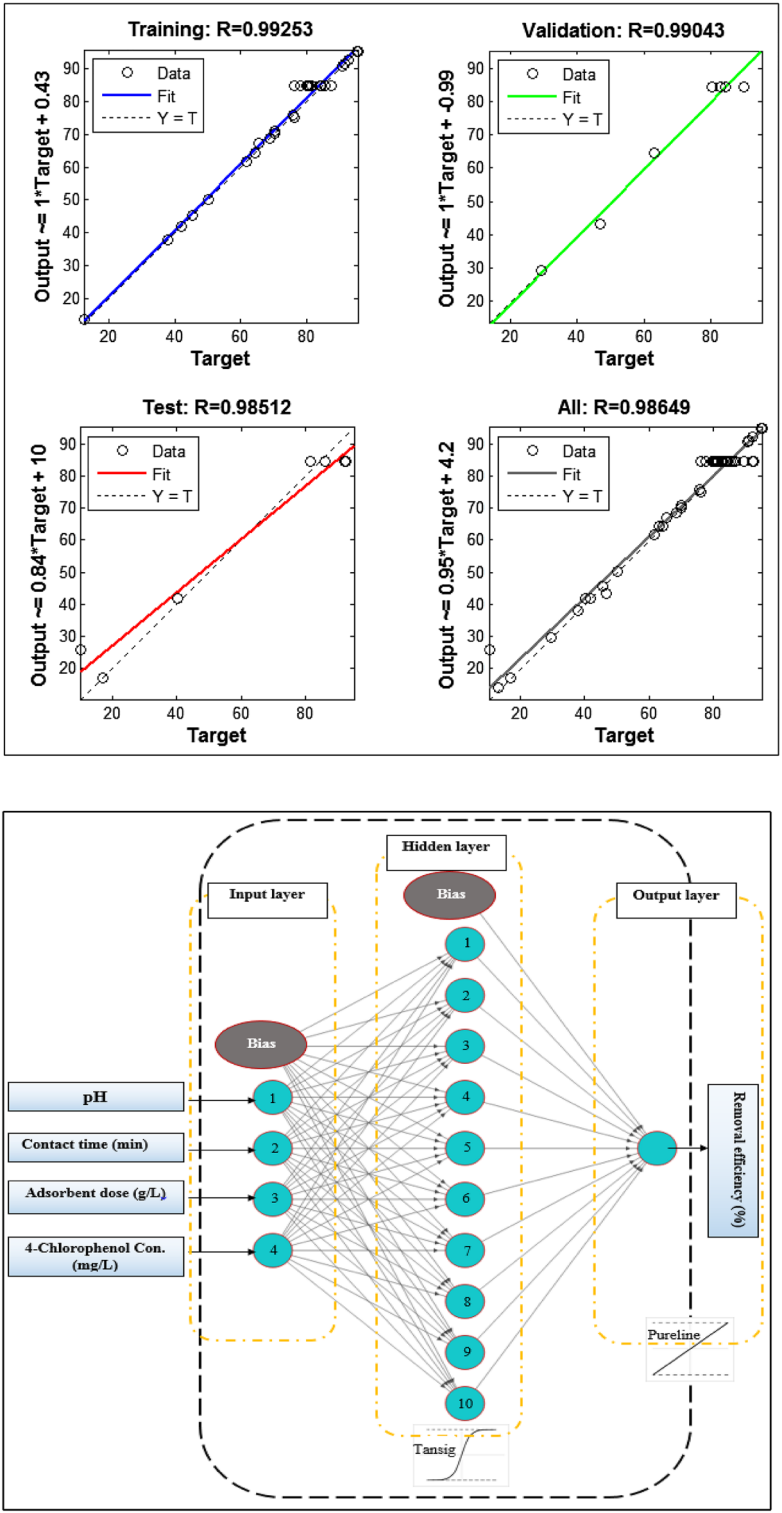
Topology and performance of the ANN model for training, validation, testing, and all data.

The ANN model was used to predict removal efficiency according to the data in Table 4. Operational variables and adsorption efficiency were used as the inputs and outputs of the ANN model. MSE and R^2^ statistical metrics vary by changes in the number of neurons in the hidden layer. MSE and R^2^ parameters showed the lowest and largest values, respectively, when the number of neurons in the hidden layer was set at 10. The topology and performance of the ANN model for training, validation, testing, and all data are presented in Fig. 4.

ANN model to predict response establishes the mathematical equation expressed as follows:

9 $${Y}_{n}= {f}_{0}\left\{{b}_{0}+\sum_{k=1}^{h}\left[{w}_{k}\times {f}_{h}({b}_{hk}+\sum_{i=1}^{m}{w}_{ik}{X}_{ni})\right]\right\} .$$

The ANN model is initially evaluated by testing data. The subplot related to testing data confirms a good fitness between predictions and experimental data with R^2^ equal to 0.9925. The model’s efficiency was further assessed by validating and testing data, and outcomes illustrate that the model predictions are reasonably close to the experimental data with R^2^ of 0.9904 and 0.9851, respectively. These results generally revealed that the ANN approach shows a higher compatibility for modeling the adsorption process than the RSM-CCD method.

**Figure 5:**
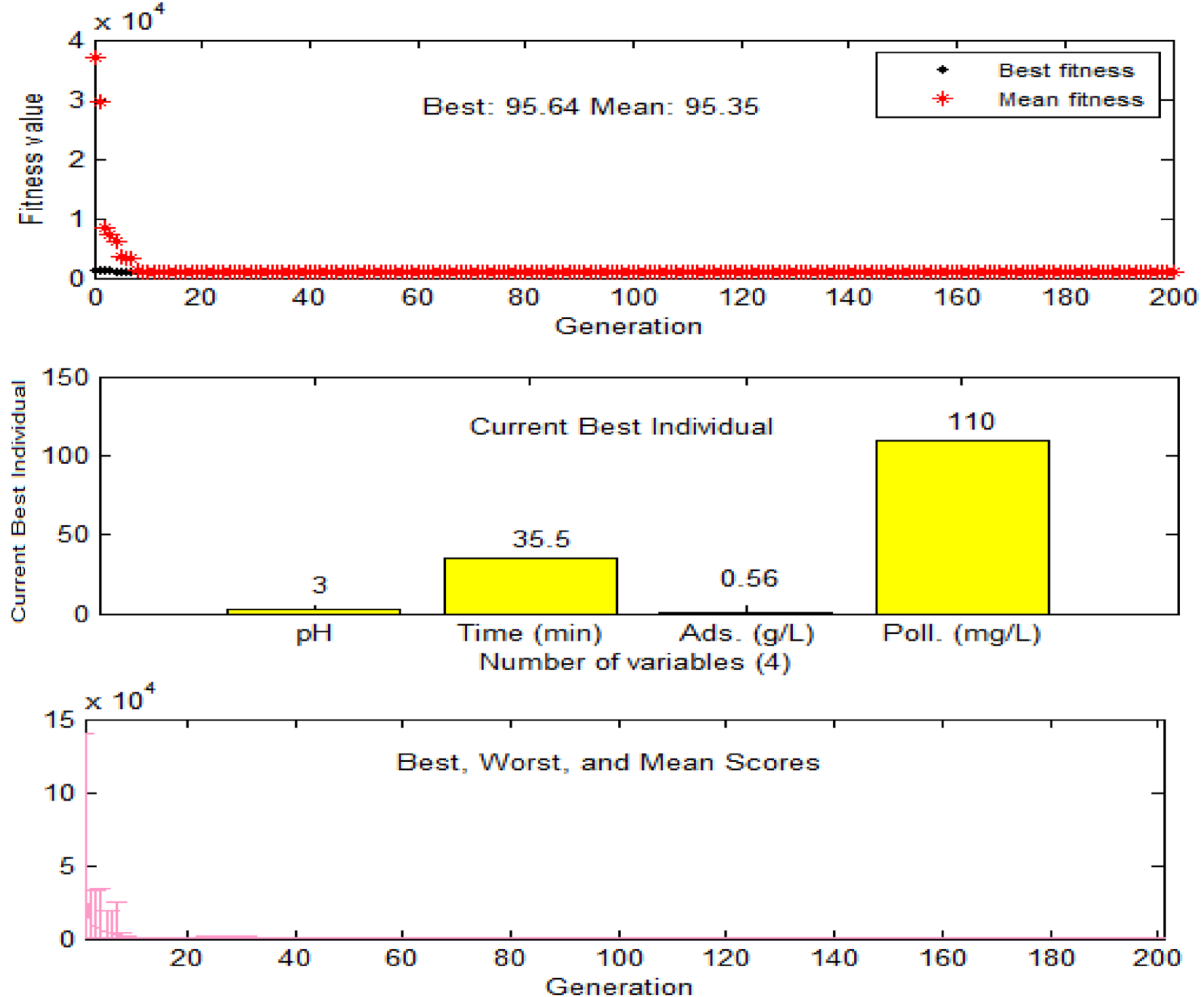
Optimization results of the process parameters based on the ANN-GA approach.

According to the appropriateness of the RSM model, the points that optimize the removal rate of 4-chlorophenol are equal to pH of 3, Time of 35 min, 4-cp concentration of 110 mg/L, Adsorbent dose of 0.55 g/L and the maximum removal efficiency was 95.352% (Table 2). The ANN-GA approach (Fig. 5) found almost the same results**,** where the maximum removal efficiency of 95.64% was obtained at pH of 3, Time of 35.5 min, 4-cp concentration of 110 mg/L, Adsorbent dose of 0.56 g/L.

### The effects of experimental parameters

#### Effect of contact time and 4-chlorophenol concentration

**Figure 6:**
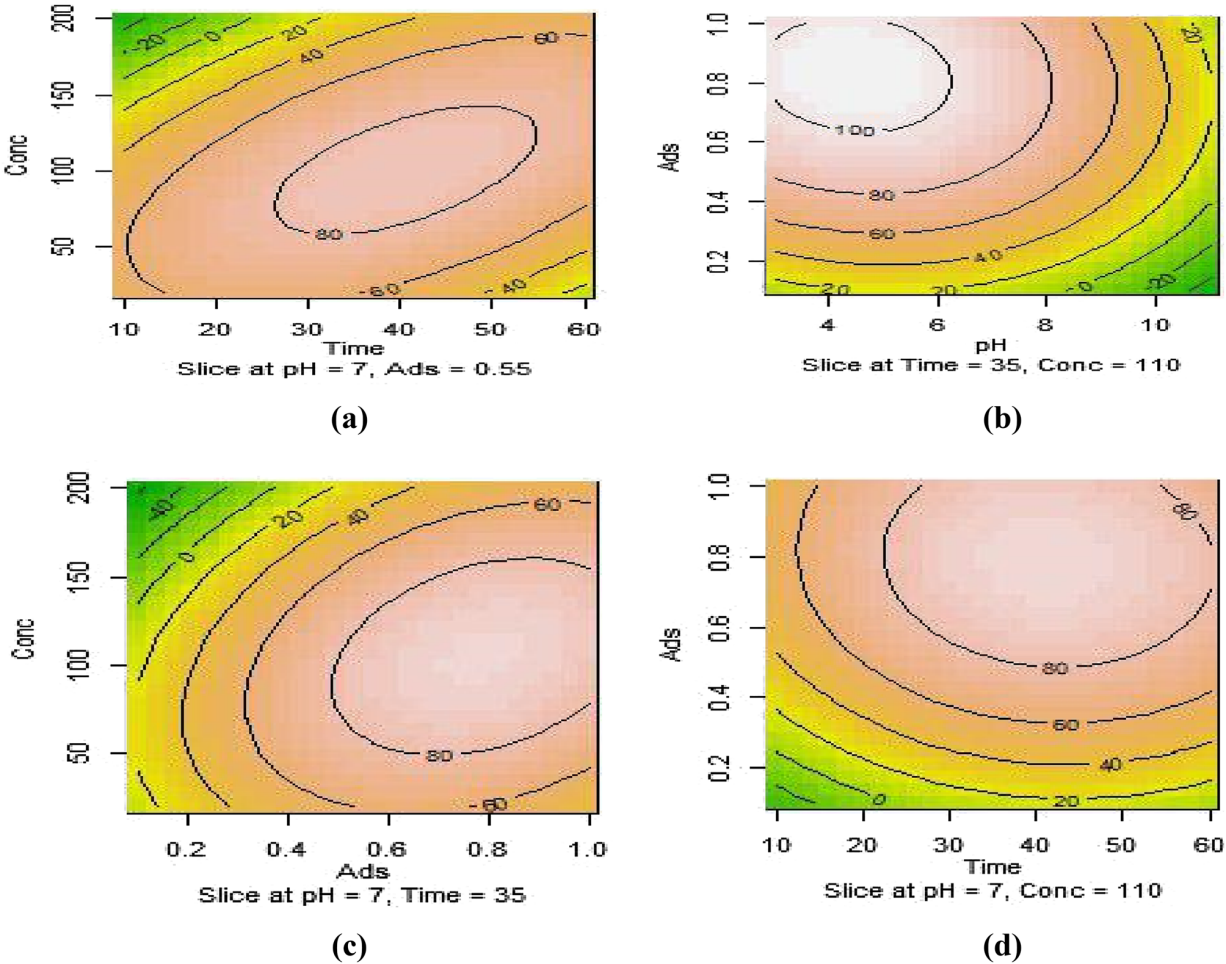
Contour plots and the main effect of studied variables (**a**) Contact time and 4-chlorophenol concentration. (**b**) pH and adsorbent dose. (**c**) Adsorbent dose and 4-chlorophenol concentration. (**d**) Contact time and adsorbent dose.

Figure 6a shows the interaction effect of contact time and 4-chlorophenol concentration on adsorbate removal efficiency. This counter curve shows the interaction effects of the variables of contact time and 4-chlorophenol concentration at a pH of 7 and an adsorbent amount of 0.55 g/L. As can be seen, increasing the contact time (up to about 40 min) and when the concentration of 4-chlorophenol increased to 100, the removal efficiency increases; within 45 min and the concentration of 4-chlorophenol equal to 110 mg/L, the removal efficiency of 4-chlorophenol reached over than 90%, which can be due to the high dependence of absorption 4-Chlorophenol is mainly amine groups on the MAC surface^23^.

#### Effect of pH and adsorbent dose

Figure 6b shows the contour curve of 4-chlorophenol Removal with changes in pH and adsorbent dose. This curve shows the effects of pH variables and adsorbent dosage at a constant time of 35 min, and the initial concentration of 4-chlorophenol was 110 mg/L. In general, pH has an influential role in removing 4-chlorophenol in the adsorption process due to its effect on the functional groups of the adsorbent surface. The highest removal efficiency of 4-chlorophenol occurs at a pH of about 3; at a pH higher than 8, the removal efficiency decreases. The increased 4-chlorophenol may be due to the competition between hydroxide ions and 4-chlorophenol in exchange and separation, which are related to functional groups on the absorbent surface^38^. On the other hand, with the increase in the amount of adsorbent, the removal efficiency increases due to the increase in the specific area of the adsorbent and available free adsorption sites^39,40^.

#### Effect of adsorbent dose and 4-chlorophenol concentration

Figure 6c shows the contour curve of the adsorbent dose and 4-chlorophenol concentration interaction effect. This curve shows the effects of adsorbent dosage variables and 4-chlorophenol concentration at a fixed time of 35 min and pH equal to 7. As can be seen, when the initial concentration of 4-chlorophenol increases with an increasing amount of adsorbent, the highest absorption efficiency can be obtained; at the initial concentration of 50 mg/L, the removal efficiency increases with the increase of the adsorbent dose. According to the contour curve and the negative coefficient of 4-chlorophenol, the concentration has an inverse effect on the removal efficiency. The reduction in removal efficiency at high concentrations of 4-chlorophenols can be due to decreases in the available surface of the adsorbent to the number of moles of the pollutant. Therefore, the higher removal of 4-chlorophenol at lower concentrations of 4-chlorophenol is due to the sufficient free adsorption sites at low concentrations of the adsorbent^41,42^.

#### Effect of contact time and adsorbent dose

Figure 6d shows the contour curve of the interaction effects of contact time and adsorbent dose variables on the removal efficiency of 4-chlorophenol. This curve shows the interaction effects of contact time and adsorbent dose variables at pH equal to 7 and the initial concentration of 4-chlorophenol of 110 mg/L. Increasing the contact time of the pollutant with the adsorbent by increasing the amount of adsorbent led to higher removal efficiency of 4-chlorophenol. In the contact time of fewer than 45 min, it was observed that over 90% removal of 4-chlorophenol; in More than 45 min, the pollutant removal rate is almost constant.

### Isotherms

Adsorption isotherms are commonly used to describe how the adsorbent reacts with the adsorbate and play an essential role in optimizing the adsorbent consumption^16,43^. The adsorption isotherms have been investigated to obtain adsorption capacity and investigate the adsorption behaviour of 4-chlorophenol using powdered activated carbon modified with amine groups (MAC). Langmuir, Freundlich, and Temkin isotherms models have been employed in this study. The fitted parameters achieved from the linear and non-linear forms of isotherm models are listed in Table 8.

**Table 8:** Constants and parameters of isotherm models.

Isotherm	Parameters	Values	
		Linear	Non-linear
Langmuir	K_L_ (mg/g)	0.125	0.135
	q_m_ (mg/g)	316.1	305.502
	R_L_	0.674	0.521
	R^2^	0.993	0.998
Freundlich	K_f_ (mg/g)	76.29	80.16
	1/n	0.316	0.294
	R^2^	0.967	0.974
Temkin	b_T_	52.96	46.774
	A_T_ (L/g)	2.537	2.871
	R^2^	0.943	0.962

#### Langmuir isotherm

**Figure 7:**
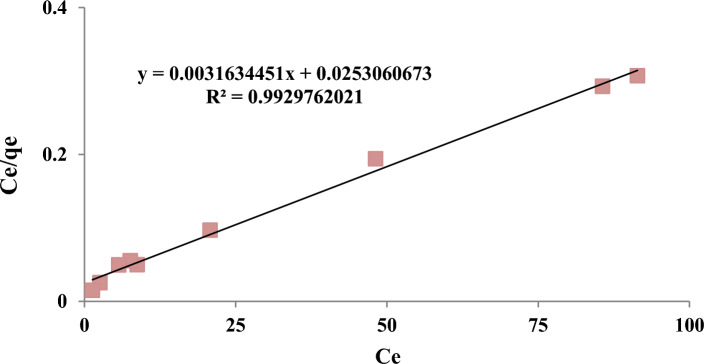
Langmuir isotherm model in 4-chlorophenol Removal with MAC.

**Figure 8:**
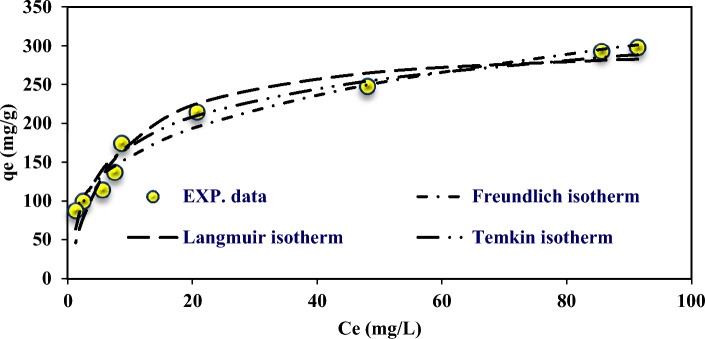
Non-linear Langmuir, Freundlich and Temkin isotherm models in 4-chlorophenol Removal with MAC.

According to Table 8, the fitted data with isotherm models were as follows: Langmuir < Freundlich < Temkin. R^2^ as a statistical metric also verifies that experimental data were better fitted to non-linear form isotherm models than linear form. Langmuir adsorption model is used with the assumption of single-layer adsorption on the surface of absorbent material with limited and identical adsorption sites^44^. The results showed that the absorption of 4-chlorophenols has good compliance with Langmuir isotherms (R^2^ > 0.95). Figures 7 and 8 show the Langmuir adsorption isotherm model in removing 4-chlorophenols using powdered activated carbon modified with amine groups (MAC).

One of the essential parameters in the Langmuir equation is the separation coefficient (R_L_), indicating the adsorbent’s tendency to separate and remove the adsorbate. The data obtained from the Langmuir isotherm showed that the R_L_ for the adsorbent is in the optimal range (0–1) (Table 4). The authors reported that the maximum absorption capacity of activated carbon for 4-chlorophenols was 186.68 mg/g. The maximum adsorption capacity of synthesized MAC was 316.1 mg/g, indicating the significantly increased adsorption capacity due to surface modification with the amine group^45^. The authors^16^ surveyed the removal of the phenolic compound 4,2 dichlorophenol by activated carbon and activated carbon modified with amine groups. The authors reported that the maximum absorption capacity was 285.71 mg/g, which is almost close to the results of the present study. In addition, it showed that the maximum adsorption capacity of activated carbon modified with amine groups was 198.66 mg/g, which is lower than the present study^37^.

#### Freundlich isotherm

**Figure 9:**
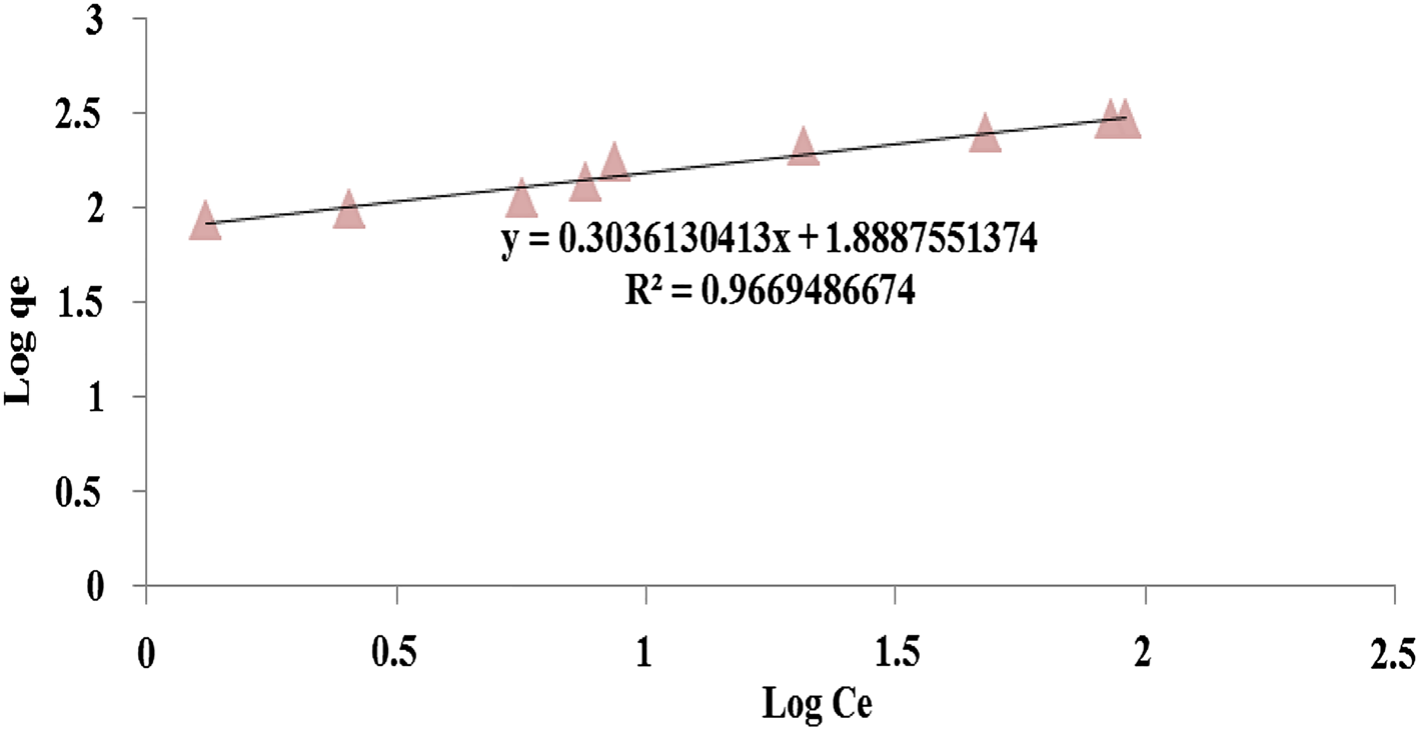
Freundlich isotherm model in 4-chlorophenol Removal with MAC.

The Freundlich model is an empirical equation based on the solute distribution between the solid and aqueous phases at equilibrium. This experimental equation describes the adsorption process based on the assumption that the adsorbent has a heterogeneous surface with different classes of adsorption sites. According to Table 8, the adsorption capacity (K_f_) for the modified activated carbon was 76.29 and 80.19 mg/g for linear and non-linear Freundlich isotherm form, indicating a more reactive site on MAC due to modification with amine groups. Amine groups cause changes such as a significant increase in surface area, an increase in the diameter of the pores, and a change in the three-dimensional shape of the adsorbent structure. Therefore, this structural change also causes an increase in the absorption sites and consequently increases the absorption of 4-chlorophenol^32^. Figure 9 shows the Freundlich adsorption isotherm model in removing 4-chlorophenols using powdered activated carbon modified with amine groups (MAC).

One of the parameters in the Freundlich isotherm is 1/n, which shows the adsorption intensity. Given that the calculated absorption intensity is in the desired range (0–1), it can be concluded that modifying the activated carbon by amino groups with more active sites led to higher adsorption capacity. As the value of 1/n tends to zero, this interaction is more potent and stronger^46^. In the study on modified pumice with cationic surfactant for removal of 4-chlorophenol, the authors reported that the Freundlich isotherm was well-fitted to describe the equilibrium relationships^47^.

#### Temkin isotherm

**Figure 10:**
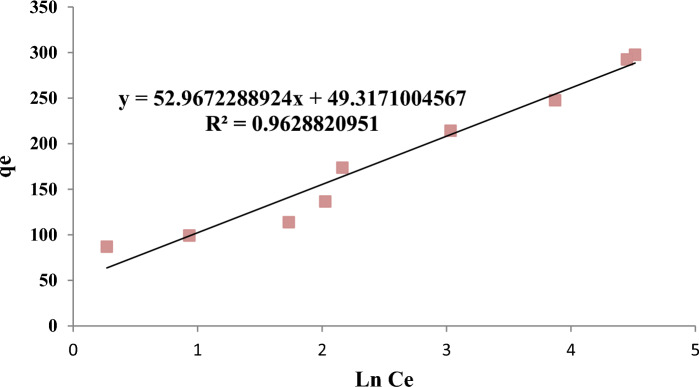
Temkin isotherm model in 4-chlorophenol Removal with MAC.

Analysis of the isotherm data is important to develop an equation that correctly represents the results and could be used for design purposes. Figure 10 shows the isotherm model of Temkin adsorption in removing 4-chlorophenols using powdered activated carbon modified with amine groups (MAC).

Temkin isotherm expresses the indirect effect of interaction between adsorbent/adsorbate on the adsorption process^48^. In this isotherm, it is assumed that the adsorption temperature of all molecules decreases linearly with the increase of the covered surface; the absorption process is determined by the uniform distribution of binding energies on the absorbent surface^49^.

#### Kinetics

Adsorption kinetics is used to investigate the absorption mechanism. This study’s data were fitted with pseudo-first-order, pseudo-second-order, Elovich, and particle diffusion kinetics (Table 9).

**Table 9:** Constants and parameters of the kinetic models.

Kinetic model	Parameters	Linear value	Non-linear value
Pseudo-first-order	k_1_	0.106	0.114
	q_e_	145.49	147.29
	R^2^	0.896	0.934
Pseudo-second-order	k_2_	0.001	0.0014
	q_e_	163.397	168.81
	R^2^	0.996	0.998
Elovic	β (g/mg)	0.034	0.036
	α (mg/(g min))	95.892	96.14
	R^2^	0.886	0.917
Intra particle	K_dif_ (mg/(g min^0.5^))	10.725	19.08
	C (mg/g)	72.372	75.17
	R^2^	0.793	0.887

**Figure 11:**
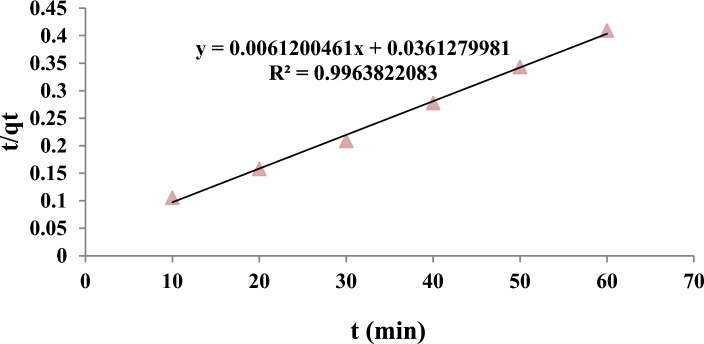
The pseudo quadratic kinetic model curve in 4-chlorophenol Removal with MAC.

**Figure 12:**
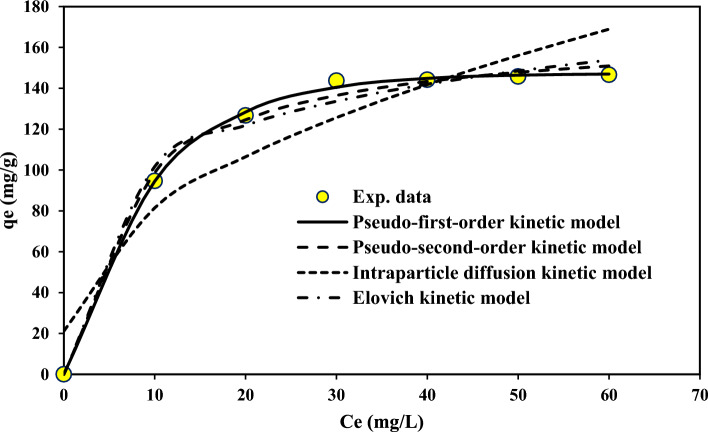
The pseudo-first order, pseudo-second order, intraparticle diffusion kinetic and elovich kinetic model curve in 4-chlorophenol Removal with MAC.

As can be seen in Table 9, the regression coefficient in the pseudo-second-order kinetic model in both the linear and non-linear forms is higher compared to pseudo-first-order, Elovich, and intraparticle models. The adsorption process follows the pseudo-quadratic model (R^2^ = 0.996 (linear form and R^2^ = 0.998)). The pseudo quadratic kinetic model curve can be seen in Figs. 11 and 12. The Qe value of 163.397 mg/g reported in Table 9 represents the equilibrium adsorption capacity obtained from the pseudo-second-order kinetic model. In comparison, the Qm value of 316.1 mg/g reported in Table 8 represents the maximum adsorption capacity obtained from the Langmuir isotherm model. The pseudo-second-order kinetic model and the Langmuir isotherm model describe different aspects of the adsorption mechanism and the adsorption capacity of the adsorbent. The pseudo-second-order kinetic model assumes that the rate-limiting step is chemisorption, while the Langmuir isotherm model assumes that the adsorption occurs on a homogeneous surface with a finite number of identical sites. The maximum adsorption capacity obtained from the Langmuir isotherm model represents the theoretical maximum amount of pollutant that can be adsorbed onto the adsorbent at saturation, and it is a function of the affinity between the adsorbent and the adsorbate^50^. Both models can obtain important information about the adsorption mechanism and the adsorption capacity of the adsorbent. They should be interpreted in the context of the specific experimental conditions and the properties of the adsorbent and the adsorbate^51^.

The pseudo-second-order model's correlation coefficient (R^2^) indicated that the pseudo-second-order model than the other models better fitted the adsorption of the 4-chlorophenol Removal with MAC. Pseudo-quadratic kinetics shows that the adsorbent concentration and adsorption capacity significantly affect the reaction's kinetics. Yang et al. reported that kinetic studies of activated carbon modified with aminated groups showed that the adsorption process follows a pseudo-quadratic model, consistent with our study of activated carbon modified with amine groups^24^.

### Thermodynamics

By fitting the data, thermodynamic parameters and constants for the adsorption of 4-chlorophenol onto MAC were achieved and summarized in Table 10. The negative value of ∆H^0^ parameter indicates the exothermic nature of 4-chlorophenol adsorption onto MAC. The negative value of ∆G^0^ exhibits the feasibility of the adsorption process and its spontaneous nature^52^. Lastly, the negative value of ∆S^0^ describes decreased randomness during adsorption^53^.

**Table 10:** Thermodynamic parameters for adsorption of 4-chlorophenol onto MAC.

Adsorbent	$\Delta G({\text{kJ/mol}}^{-1})$					${\Delta H}^{^\circ } ({\text{kJ/mol}}^{-1})$	$\Delta {S}^{^\circ }({\text{J/mol}}^{-1} \, {\text{K}}^{-1})$	R^2^
	288 °K	298 °K	308 °K	318 °K	328 °K			
MAC	− 6.7	− 5.9	− 5.2	− 4.5	− 3.1	− 28.3	− 66.3	0.9882

### Effect of co-existing ions

**Figure 13:**
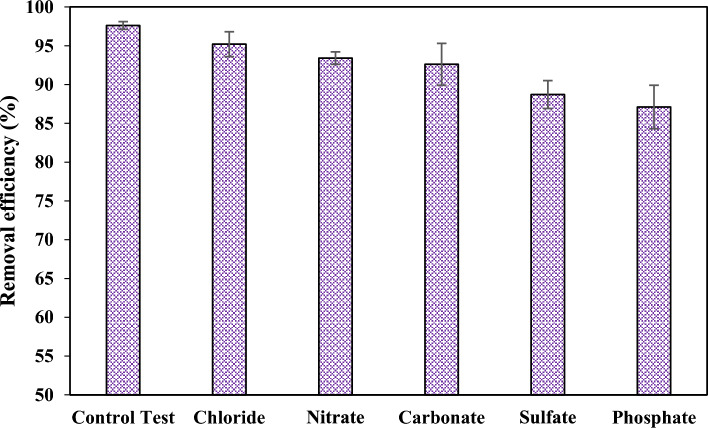
Effect of co-existing anions on the adsorption performance.

The presence of interfering ions in water matrices is inevitable, and these ions will likely affect the adsorption process. Therefore, some common anions, including chloride, carbonate, sulfate, nitrate, and phosphate, were added individually to the reaction solution to prepare 50 mmol/L concentrations. Figure 13 elucidates the effect of these anions on the adsorption performance. It is observed that the inhibition effect increases in the order of chloride < nitrate < carbonate < sulfate < phosphate. Nonetheless, the efficiency decline is not intense and demonstrates that the adsorbent maintains its adsorption capacity in the presence of the interfering ions. Also, the findings reveal that the anions with the more negative charge density, such as sulfate and phosphate, are more effective in lowering the removal efficiency.

### Reusability test

**Figure 14:**
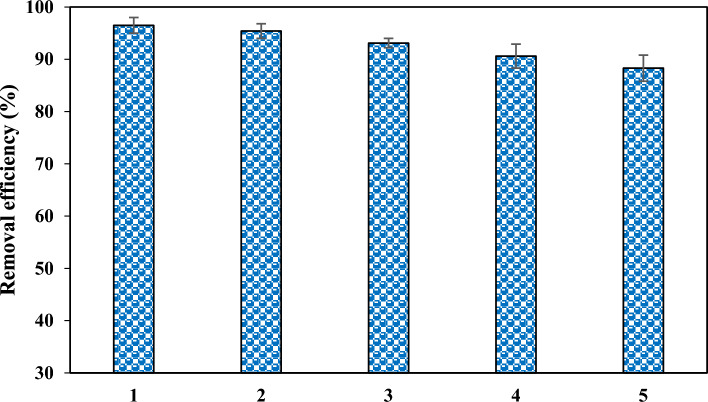
Recyclability examination of MAC for five consecutive cycles.

The adsorbent’s reusability is quite important from an economic point of view. To this end, the adsorption efficiency was examined for five successive cycles, and after each cycle, the adsorbent was isolated and washed several times with NaOH and deionized water. As shown in Fig. 14, the results indicated that the uptake rate dropped only about 6% after five cycles. This drop is not notable and interestingly highlights the desirable reusability potential of MAC.

### Comparison of the present study with other reported adsorbents

To justify its viability as an effective adsorbent for 4-CP removal, the adsorption capacity of MAC on 4-CP needs to be compared to that of other adsorbents. The 4-chlorophenol adsorption capacity of this study was compared with other adsorbents reported by other researchers. Several studies have investigated the removal of phenolic compounds from water by several types of activated carbons modified with different chemical agents, and their maximum adsorption capacities (Q_max_) are listed in Table 11.

**Table 11:** Comparison of maximum adsorption capacity of 4-chlorophenol between various adsorbents.

	Adsorbents	C_0_ (mg/L)	pH	Dosage (g/L)	Isotherm	Kinetic	Q_m_ (mg/g)	Removal percentage (%)	Ref.
1	NaOH-treated CSBAC	100	2	6	Langmuir (R^2^: 0.9908)	Pseudo-second-order (R^2^: 0.9924)	85.79	91	^54^
2	HNO_3_-oxidized CSBAC	100	2	6	Langmuir (R^2^: 0.9844)	Pseudo-second-order (R^2^: 0.9965)	61.24	60	
3	TiO_2_-coated CSBAC	100	2	6	Langmuir (R^2^: 0.9857)	Pseudo-second-order (R^2^: 0.9981)	81.46	72.8	
4	Modified CSAC (coconut shell activated carbon)	50	2	5	Freundlich (R^2^: 0.983)	Pseudo-second-order (R^2^: 0.9981)	72.77	99.9	^55^
5	EFB ammonia-modified activated carbon	25	2	3	Langmuir (R^2^: 0.99)	Pseudo-second-order (R^2^: 0.99)	285.71	97.24	^19^
6	EFB phosphoric acid -modified activated carbon	25	2	2	Langmuir (R^2^: 0.962)	Pseudo-second-order (R^2^: 0.991)	232.56	98.11	^56^
7	Croton caudatus activated carbon	80	3	0.15	Langmuir	Pseudo-second-order	–	97.23	^57^
9	Nanosized activated carbons (bamboo and trees)	100	7	0.2	Freundlich (R^2^:0.989)	Pseudo-second-order	220.6	80	^58^
10	Nanosized activated carbons (coconut shell charcoal)	100	7	0.2	Freundlich (R^2^:0.988)	Pseudo-second-order	211.7	80	^58^
11	Red mud	60	5	10	Langmuir (R^2^: 0.99)	Pseudo-second-order	36.2	97	^59^
12	Present study	110	3	0.55	Freundlich (R^2^: 0.967)	Pseudo-second-order (R^2^: 0.996)	316.1	95.352	-

Comparisons are made regarding adsorption capacity (mg/g), optimum pH, initial concentration (mg/L), removal percentage, isotherms, and kinetics. The table shows that materials such as EFB ammonia-modified activated carbon and EFB phosphoric acid-modified activated carbon show higher adsorption capacities to other adsorbents such as NaOH-treated CSBAC, croton caudatus, and red mud activated carbon. It is also observed that the modified activated carbon with amine groups used in this study (3136.1 mg/g) has demonstrated a better adsorption capacity than similar materials.

## Conclusion

This study investigated the absorption process of 4-chlorophenols by activated carbon modified with amino groups. The CCD experiment design showed the interaction effects between pH parameters, adsorbent dose, contact time, and initial pollutant concentration. The XRD, FTIR, and SEM analyses showed that powdered activated carbon’s surface modification was well done. The model results showed that the initial concentration of 4-chlorophenol has opposite effects on its removal, and the effect of variables of contact time and adsorbent dose have a direct relationship with the amount of 4-chlorophenol Removal. In addition, at a pH lower than 8, the removal efficiency of 4-chlorophenol with activated carbon modified with amine groups was high. The modified adsorbent can remove pollutants in a wide range of pH. The removal of 4-chlorophenols using activated carbon modified with amine groups follows the Langmuir isotherm model and pseudo-second-order kinetics. The maximum adsorption capacity for removing 4-chlorophenols by activated carbon modified with amine groups was 316.1 mg/g. The thermodynamic study indicated that the adsorption process is exothermic and spontaneous. The interfering anions showed no drastic negative effect on removal efficiency, and the reusability test exhibited an excellent adsorption efficiency of MAC even after five successive cycles. This study showed that amine groups increase the removal efficiency by increasing the adsorbent's surface area and active adsorption sites. The modification of the adsorbent's surface increases the adsorbent’s maximum capacity.

It should be noted that there is a need for further research to validate the findings of the study using real samples and to optimize the adsorption process for different types of organic pollutants. This would provide a more comprehensive understanding of the potential applications of amine-functionalized activated carbon for removing 4-chlorophenol from aqueous environments. Also, the stirring rate is an important variable that can significantly impact adsorption. Future studies should carefully consider the effect of stirring rate on the adsorption process and optimize this variable to maximize the adsorption capacity.

## Data Availability

All data generated or analyzed during this study are available from the corresponding author upon reasonable request.
